# Supplementary material for: Prediction model for in-hospital mortality in patients at high altitudes with ARDS due to COVID-19
Source: PLoS One. 2023 Oct 26;18(10):e0293476. doi: 10.1371/journal.pone.0293476 (PMC10602283; doi:10.1371/journal.pone.0293476)
Supplement: S1 File — (DOCX) [file pone.0293476.s001.docx]

**Supplementary material**

**Adjusted P/F prediction model**

When adjusting the P/F as recommended by Berlin, out of 2,210, 2,042 patients remained. That is, 168 patients in the total cohort did not meet the definition of ARDS. In other words, the incidence of ARDS decreased 7.6% when adjusting the P/F.

The model is entered the variables with less than 10% missing data and with p <0.25 in the univariate logistic regression (Table 1 supplementary material).

**Table 1 supplementary material. Univariate logistic regression analyses.**

| Variable | OR | 95% CI | p | Missing | (%) |
| --- | --- | --- | --- | --- | --- |
| Age | 1.0490 | 1.0415-1.0566 | 0.000 | 0 | 0 |
| Weight | 0.9883 | 0.9822-0.9944 | 0.000 | 0 | 0 |
| Height | 0.9711 | 0.9611-0.9812 | 0.000 | 0 | 0 |
| Leukocyte count | 1.0011 | 0.9995-1.0129 | 0.854 | 10 | 0.48 |
| Neutrophil count | 0.9949 | 0.9758-1.0143 | 0.601 | 11 | 0.53 |
| Lymphocyte count | 0.9344 | 0.8511-1.0259 | 0.155 | 10 | 0.48 |
| Monocyte count | 1.0970 | 0.8915-1.3498 | 0.382 | 10 | 0.48 |
| Eosinophil count | 1.6456 | 0.4809-5.6313 | 0.427 | 11 | 0.53 |
| Basophil count | 0.0872 | 0.0024-3.2192 | 0.185 | 10 | 0.48 |
| Haematocrit | 0.9769 | 0.9646-0.9894 | 0.000 | 9 | 0.44 |
| Haemoglobin | 0.9184 | 0.8853-0.9527 | 0.000 | 9 | 0.44 |
| Platelet count | 0.9969 | 0.9960-0.9979 | 0.000 | 15 | 0.73 |
| Neutrophil/lymphocyte ratio | 1.0188 | 1.0099-1.0277 | 0.000 | 11 | 0.53 |
| Creatinine | 1.4292 | 1.0876-1.2143 | 0.000 | 12 | 0.58 |
| D-dimer | 1.0000 | 1.0000-1.0000 | 0.438 | 117 | 5.72 |
| Lactate dehydrogenase | 1.0012 | 1.0008-1.0017 | 0.000 | 71 | 3.47 |
| C-reactive protein | 0.9998 | 0.9989-1.0007 | 0.641 | 173 | 8.47 |
| Sodium | 0.9693 | 0.9529-09859 | 0.000 | 139 | 6.80 |
| Potassium | 1.2627 | 1.1090-1.4377 | 0.000 | 137 | 6.70 |
| Respiratory rate | 1.0050 | 0.9856-1.0248 | 0.617 | 0 | 0 |
| Heart rate | 0.9945 | 0.9900-0.9990 | 0.016 | 0 | 0 |
| Systolic pressure | 0.99844 | 0.9946-1.0023 | 0.429 | 0 | 0 |
| Diastolic pressure | 0.9916 | 0.9848-0.9984 | 0.016 | 0 | 0 |
| Body mass index | 0.9970 | 0.9805-1.0138 | 0.723 | 0 | 0 |
| Charlson comorbidity index | 1.4603 | 1.3719-1.5544 | 0.000 | 1 | 0.04 |
| P/F diagnosis | 0.9895 | 0.9977-0.9913 | 0.000 | 0 | 0 |
| Female sex | Reference: |  |  | 0 | 0 |
| Male sex | 0.8224 | 0.6852-0.9070 | 0.035 | 0 | 0 |
| Mild ARDS | Reference: |  |  | 0 | 0 |
| Moderate ARDS | 1.9523 | 1.6151-2.3599 | 0.000 | 0 | 0 |
| Severe ARDS | 4.3903 | 3.2181-5.9896 | 0.000 | 0 | 0 |
| High blood pressure | 1.0530 | 0.8631-1.2846 | 0.611 | 0 | 0 |
| Variables with more than 10% of data lost | | | |  |  |
| Ferritin | 1.0002 | 1.0001-1.0003 | 0.002 | 1565 | 76.6 |
| Procalcitonin | 1.0382 | 1.0166-1.0602 | 0.000 | 995 | 48.7 |
| Chlorine | 0.9801 | 0.9642-0.9962 | 0.016 | 233 | 11.4 |
| Calcium | 0.5454 | 0.4544-0.6454 | 0.000 | 735 | 35.9 |
| Glucose | 1.0023 | 1.0009-1.0037 | 0.001 | 1065 | 52.1 |
| Albumin | 0.2788 | 0.0954-0.8148 | 0.020 | 1959 | 95.9 |
| Alkaline phosphatase | 1.0005 | 0.9992-1.0018 | 0.419 | 1581 | 77.4 |
| Alanine aminotransferase | 1.0002 | 0.9997-1.0008 | 0.401 | 208 | 10.1 |
| Aspartate aminotransferase | 1.0008 | 1.0001-1.0015 | 0.024 | 209 | 10.2 |
| Total bilirubin | 1.0920 | 0.9631-1.2381 | 0.170 | 209 | 10.2 |
| Variables not considered to be entered in the model | | | |  |  |
| P/F at admission | 0.9952 | 0.9940-0.9964 | 0.000 | 0 | 0 |
| Lower P/F | 0.9757 | 0.9729-0.9885 | 0.000 | 0 | 0 |
| Invasive mechanical ventilation | 5.3826 | 4.4508-6.5096 | 0.000 | 0 | 0 |

Univariate analysis by mortality. P/F adjusted.

Table 2 supplementary material shows the coefficients with their 95% CIs both for patients with complete data and after data imputation. Bootstrapping was performed, and the coefficients remained unchanged compared to the original analysis with and without data imputation.

**Table 2 supplementary material. Final model after step-by-step selection of the covariates.**

| Covariate | Imputed data model | Model with complete data^a^ | Bootstrapping |
| --- | --- | --- | --- |
| Age | 0.0474 (0.0394, 0.0554) | 0.0457 (0.0371, 0.0543) | 0.0473 (0.0388, 0.0545) |
| Weight | Not significant | 0.0062 (-0.0021, 0.0145) | Not significant |
| Height | -0.0127 (-0.0247, -0.0007) | -0.0155 (-0.0290, -0.0020) | -0.01268 (-0.0251, 0.0000) |
| Haemoglobin | -0.0711 (-0.1154, -0.0267) | -0.0628 (-0.1100, -0.0156) | -0.0710 (-0.1221, -0.0185) |
| Platelets | -0.0034 (-0.0044, -0.0023) | -0.0034 (-0.0045, -0.0023) | -0.0033 (-0.0045, -0.0022) |
| Creatinine | 0.0972 (0.0379, 0.1566) | 0.1009 (0.0384, 0.1633) | 0.0972 (0.0305, 0.1596) |
| Lactate dehydrogenase | 0.0012 (0.0007, 0.0017) | 0.0012 (0.0007, 0.0018) | 0.0011 (0.0005, 0.0018) |
| Sodium | -0.0198 (-0.0378, -0.0019) | -0.0237 (-0.0432, -0.0042) | -0.0198 (-0.0385, -0.0013) |
| Potassium | 0.1879 (0.0353, 0.3406) | 0.1800 (0.0202, 0.3397) | 0.1879 (0.036, 0.324) |
| Moderate ARDS | 0.7100 (1.0489, 1.7246) | 0.6058 (0.3885, 0.8232) | 0.7100 (0.4949, 0.9190) |
| Severe ARDS | 1.3867 (1.1376, 1.6966) | 1.2420 (0.8931, 1.5909) | 1.3867 (1.053, 1.705) |

The final model coefficients with their 95% CIs are reported with complete data after data imputation and bootstrapping.

^a^1,832 patients with complete data.

The linearity assumption is fulfilled. MFPs compliance does not require polynomial adjustment of the variables involved.


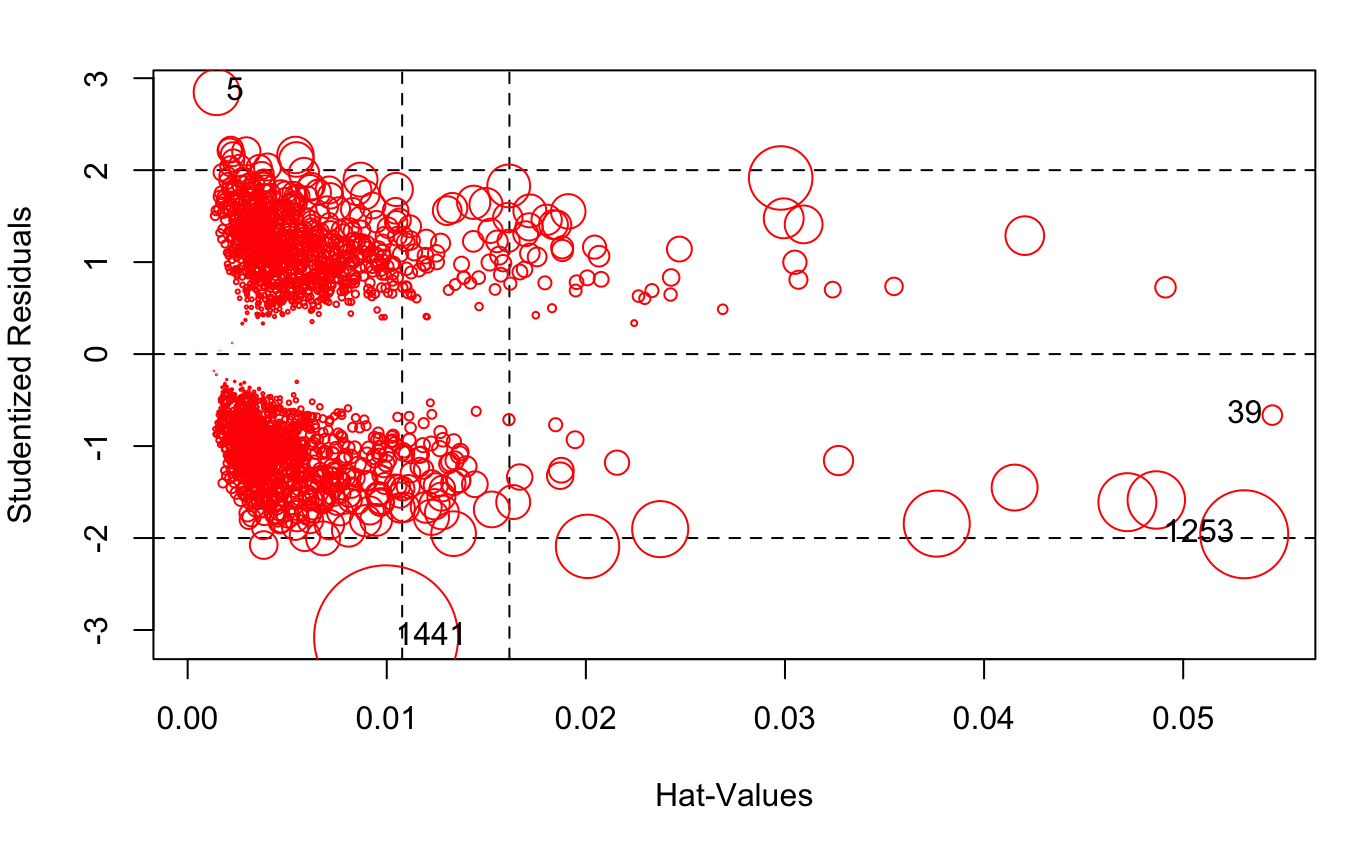


**Fig 1 supplementary material. Graph of studentized residuals versus hat values.**

There are four influential measures: 5, 39, 1253, 1441.
