# Supplementary material for: Prediction model for in-hospital mortality in patients at high altitudes with ARDS due to COVID-19
Source: PLoS One. 2023 Oct 26;18(10):e0293476. doi: 10.1371/journal.pone.0293476 (PMC10602283; doi:10.1371/journal.pone.0293476)
Supplement: Attachment — Submitted filename: Response to Reviewers.docx [file pone.0293476.s004.docx]

Bogotá, July 2023.

Response to Reviewers

Thank you very much for reviewing this manuscript and for your observations.

PONE-D-23-14199

Prediction model for in-hospital mortality in patients at high altitudes with ARDS due to COVID-19

PLOS ONE

Journal Requirements:

When submitting your revision, we need you to address these additional requirements.

1. Please ensure that your manuscript meets PLOS ONE's style requirements, including those for file naming. The PLOS ONE style templates can be found at
   <https://journals.plos.org/plosone/s/file?id=wjVg/PLOSOne_formatting_sample_main_body.pdf>

and 
<https://journals.plos.org/plosone/s/file?id=ba62/PLOSOne_formatting_sample_title_authors_affiliations.pdf>

*All the formats for author names, affiliations, and the body of the text were followed correctly.*

2. Please review your reference list to ensure that it is complete and correct. If you have cited papers that have been retracted, please include the rationale for doing so in the manuscript text, or remove these references and replace them with relevant current references. Any changes to the reference list should be mentioned in the rebuttal letter that accompanies your revised manuscript. If you need to cite a retracted article, indicate the article’s retracted status in the References list and also include a citation and full reference for the retraction notice.

*All references were reviewed, and no retracted articles were found. An error was identified in the introduction, where references skipped from 7 to 23. This was due to a mistake with the reference manager, which did not add 5 articles. The issue has been corrected, and all references have been added and organized to ensure their proper alignment with the corresponding parts of the text. The references are formatted in Vancouver style, following the journal's recommended style.*

**Comments to the Author**

1. Is the manuscript technically sound, and do the data support the conclusions?

The manuscript must describe a technically sound piece of scientific research with data that supports the conclusions. Experiments must have been conducted rigorously, with appropriate controls, replication, and sample sizes. The conclusions must be drawn appropriately based on the data presented.

Reviewer #1: Yes

Reviewer #2: Yes

OK

2. Has the statistical analysis been performed appropriately and rigorously?

Reviewer #1: Yes

Reviewer #2: Yes

OK

3. Have the authors made all data underlying the findings in their manuscript fully available?

The [PLOS Data policy](http://www.plosone.org/static/policies.action#sharing) requires authors to make all data underlying the findings described in their manuscript fully available without restriction, with rare exception (please refer to the Data Availability Statement in the manuscript PDF file). The data should be provided as part of the manuscript or its supporting information, or deposited to a public repository. For example, in addition to summary statistics, the data points behind means, medians and variance measures should be available. If there are restrictions on publicly sharing data—e.g. participant privacy or use of data from a third party—those must be specified.

Reviewer #1: Yes

Reviewer #2: Yes

OK

4. Is the manuscript presented in an intelligible fashion and written in standard English?

PLOS ONE does not copyedit accepted manuscripts, so the language in submitted articles must be clear, correct, and unambiguous. Any typographical or grammatical errors should be corrected at revision, so please note any specific errors here.

Reviewer #1: Yes

Reviewer #2: Yes

OK

5. Review Comments to the Author

Please use the space provided to explain your answers to the questions above. You may also include additional comments for the author, including concerns about dual publication, research ethics, or publication ethics. (Please upload your review as an attachment if it exceeds 20,000 characters)

Reviewer #1: The manuscript addressed the specific situation of high altitude as a possible factor to alter the P/F predicted value for mortality, it did not show a significant difference of the corrected prediction despite another study by the author (https://intjem.biomedcentral.com/articles/10.1186/s12245-022-00426-4) from same hospital and almost same period, showed higher incidence of mortality in high altitude country of Colombia after the initial onset of disease. Can you explain this more clearly in the discussion?

In the discussion, the requested point is clarified and highlighted in yellow.

In a study published by the same research group as this work, it was demonstrated that at an altitude of 2640 masl, there exists a significant association between lower P/F ratios and in-hospital mortality[4]. However, in that previous study, the predictive capacity of the P/F ratio adjusted and unadjusted for altitude was not evaluated. Considering this aspect, the researchers hypothesized that the P/F ratio adjusted for altitude would exhibit superior predictive performance for in-hospital mortality compared to the unadjusted P/F ratio. Surprisingly, the results of the current study did not support this hypothesis, as both the adjusted and unadjusted P/F ratios showed similar predictive capabilities. These findings suggest that in patients with ARDS, the ideal cut-off point of the P/F ratio to establish vital prognosis may not be solely determined by altitude adjustments, and clinical outcomes of ARDS are influenced by multiple complex factors.

Reviewer #2: Dear authors

Many thanks for your valuable efforts. It was within the global readership interest, well written and done correctly and soundly. Availability of all data and following ethical guidelines were one of the strength points in this article.

OK

6. PLOS authors have the option to publish the peer review history of their article ([what does this mean?](https://journals.plos.org/plosone/s/editorial-and-peer-review-process#loc-peer-review-history)). If published, this will include your full peer review and any attached files.


If you choose “no”, your identity will remain anonymous but your review may still be made public.


**Do you want your identity to be public for this peer review?** For information about this choice, including consent withdrawal, please see our [Privacy Policy](https://www.plos.org/privacy-policy).

Reviewer #1: **Yes:** Dr. Hassan Aref Shabana

Reviewer #2: No

OK

*The PACE tool is used for image adjustment.*
