# Supplementary material for: Prediction model for in-hospital mortality in patients at high altitudes with ARDS due to COVID-19
Source: PLoS One. 2023 Oct 26;18(10):e0293476. doi: 10.1371/journal.pone.0293476 (PMC10602283; doi:10.1371/journal.pone.0293476)
Supplement: Attachment — Submitted filename: Rebuttal letter PONE.docx [file pone.0293476.s005.docx]

Rebuttal letter PONE-D-23-14199R1

Dear Dr. Muhammad Tarek Abdel Ghafar, M.D

Thank you for reviewing this document.

1. Please review your reference list to ensure that it is complete and correct. If you have cited papers that have been retracted, please include the rationale for doing so in the manuscript text or remove these references and replace them with relevant current references. Any changes to the reference list should be mentioned in the rebuttal letter that accompanies your revised manuscript. If you need to cite a retracted article, indicate the article’s retracted status in the References list and also include a citation and full reference for the retraction notice.
   a. All references are reviewed, they are complete, all correspond to the correct place, and no retracted articles are found.
2. Careful editing for the English language is required by a native English speaker. Please pay attention to the use of abbreviations and provide their full terms at their first mention.
   a. Sent for English editing, certificate attached.
   b. The entire document is reviewed. Abbreviations used are linked to the full term in the first mention in the abstract and also in the first mention in the manuscript. This is corrected throughout the text. Additionally, abbreviations that had been previously implemented are removed since they are not significantly repeated throughout the manuscript. This is highlighted in the manuscript using track changes.
3. Please ensure that all figures, tables, and supplementary tables and figures are cited in the text.
   a. All figures and tables are cited in the text, and this is highlighted in the manuscript using track changes.
4. Given the editing corrections in the title and abbreviations, the tables and supplementary material are added back again.
5. There are no additional comments from the peer reviewers. We are awaiting any further requirements.

David Rodriguez

Main Author
